# Conserved developmental transcriptomes in evolutionarily divergent species

**DOI:** 10.1186/gb-2010-11-3-r35

**Published:** 2010-03-17

**Authors:** Anup Parikh, Edward Roshan Miranda, Mariko Katoh-Kurasawa, Danny Fuller, Gregor Rot, Lan Zagar, Tomaz Curk, Richard Sucgang, Rui Chen, Blaz Zupan, William F Loomis, Adam Kuspa, Gad Shaulsky

**Affiliations:** 1 Department of Molecular and Human Genetics, Baylor College of Medicine, One Baylor Plaza, Houston, TX 77030, USA; 2 Graduate Program in Structural and Computational Biology and Molecular Biophysics, Baylor College of Medicine, One Baylor Plaza, Houston, TX 77030, USA; 3 Graduate Program in Developmental Biology, Baylor College of Medicine, One Baylor Plaza, Houston, TX 77030, USA; 4 Section of Cell and Developmental Biology, University of California San Diego, 9500 Gilman Drive, La Jolla, CA 92093, USA; 5 Faculty of Computer and Information Science, University of Ljubljana, Trzaska cesta 25, SI-1001 Ljubljana, Slovenia; 6 Department of Biochemistry and Molecular Biology, Baylor College of Medicine, One Baylor Plaza, Houston, TX 77030, USA

## Abstract

**Background:**

Evolutionarily divergent organisms often share developmental anatomies despite vast differences between their genome sequences. The social amoebae *Dictyostelium discoideum *and *Dictyostelium purpureum *have similar developmental morphologies although their genomes are as divergent as those of man and jawed fish.

**Results:**

Here we show that the anatomical similarities are accompanied by extensive transcriptome conservation. Using RNA sequencing we compared the abundance and developmental regulation of all the transcripts in the two species. In both species, most genes are developmentally regulated and the greatest expression changes occur during the transition from unicellularity to multicellularity. The developmental regulation of transcription is highly conserved between orthologs in the two species. In addition to timing of expression, the level of mRNA production is also conserved between orthologs and is consistent with the intuitive notion that transcript abundance correlates with the amount of protein required. Furthermore, the conservation of transcriptomes extends to cell-type specific expression.

**Conclusions:**

These findings suggest that developmental programs are remarkably conserved at the transcriptome level, considering the great evolutionary distance between the genomes. Moreover, this transcriptional conservation may be responsible for the similar developmental anatomies of *Dictyostelium discoideum *and *Dictyostelium purpureum*.

## Abstract 2

Transcriptional profiling of Dictyostelium development reveals significant conservation of transcriptional profiles between evolutionarily divergent species.

## Background

Comparisons between morphology, physiology and developmental transitions of organisms have been used for some time to study evolutionary relationships between species. We can now use genome sequence comparisons and start to relate genetic information to organismal function and morphology. High-throughput methods for the analysis of RNA, protein and metabolites are beginning to bridge the gap between genomes and functions, and evolutionary comparisons between organisms using these methods are increasing our understanding of the relationship between genes and function.

Gene regulation is sometimes surprisingly similar between divergent species, revealing common pathways in fundamental processes despite vast evolutionary distances [1,2]. Comparing the transcriptomes of evolutionarily distant organisms has revealed ancient conserved genetic networks and helped in assigning function to unknown genes [3,4]. On the other hand, there is evidence for extensive divergence of developmental gene regulation in closely related species [5] and comparative studies have shown that evolution of transcriptional regulation in specific pathways can drive divergence of developmental anatomies. For example, differences in the spatiotemporal regulation of Hox genes can account for variations in animal patterning [6] and differences in the expression patterns of conserved genes can determine variations in heart development [7]. In light of these findings, it is interesting that divergent species sometimes share developmental anatomies despite differences in their genome sequences and in their gene regulation [8]. We therefore wanted to study the global transcriptional basis of evolutionarily conserved developmental anatomies between divergent organisms.

Deep RNA sequencing (RNA-seq), in which millions of short reads are mapped to fully sequenced genomes, introduces a new dimension to transcriptome analysis. The method yields a quantitative, digital description of all the mRNA molecules in a given sample, in addition to improved sensitivity and increased dynamic range relative to hybridization based microarrays [9]. Moreover, mRNA abundance can be directly compared between genes with different sequences, within and between organisms. We used RNA-seq to compare the developmental transcriptomes of two dictyostelid species, *Dictyostelium discoideum *and *Dictyostelium purpureum*, that exhibit vast sequence divergence. The genome of *D. purpureum *has been sequenced recently and compared to that of the previously sequenced genome of *D. discoideum *(R Sucgang *et al *"Comparative genomics of the social amoeba: *Dictyostelium discoideum *and *Dictyostelium purpureum*", unpublished work). The two genomes are almost identical in size and both have a high A+T content. The genome divergence between the two species was estimated by analyzing numerous orthologous protein clusters representing plant, animal, fungal and amoebal species. This analysis suggested that the genomes of *D. discoideum *and *D. purpureum *are as different from each other as the genome of jawed fish is from that of humans (R Sucgang *et al*, unpublished work). Considering the estimate that the rates of protein evolution in the amoebozoa are comparable to those of plants and animals [10], *D. purpureum *and *D. discoideum *probably shared a common ancestor approximately 400 million years ago.

**Figure 1:**
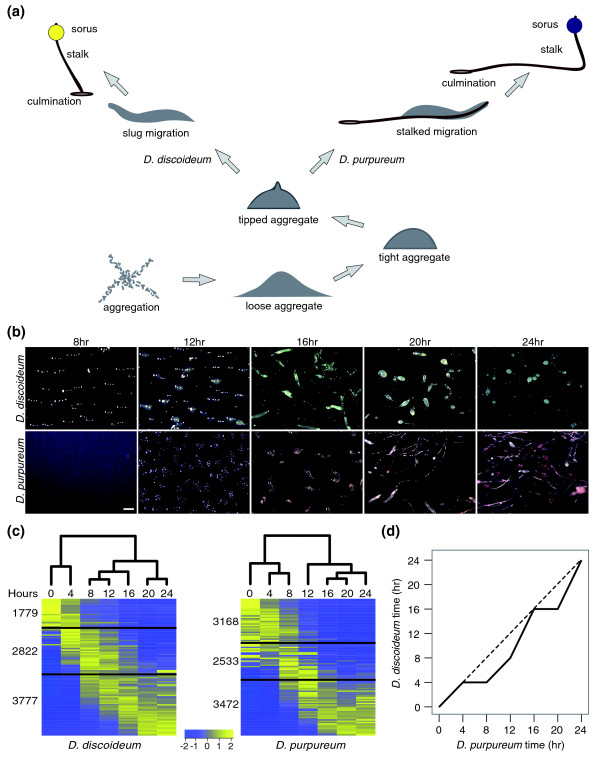
**Conservation of morphology and gene expression patterns in the developmental programs of *D. discoideum *and *D. purpureum***. **(a) **An illustration of the developmental programs. Both species begin the developmental program by aggregation of starving cells into centers that contain approximately 50,000 cells. The aggregates undergo morphological transformations from loose aggregates to tight aggregates to tipped aggregates while the cells differentiate into prespore and prestalk cells (not shown). Later in development, *D. purpureum *slugs (right) migrate while leaving a cellular stalk behind them whereas *D. discoideum *slugs do not. After culmination, the fruiting bodies are similar in size and shape and both consist of a ball of spores (sorus) carried on top of a cellular stalk as indicated. They differ in that *D. purpureum *fruiting bodies lack a basal disc at the bottom of the stalk and their sori are purple rather than yellow. **(b) **Developmental morphologies. A top view with light microscopy of cells developing on dark nitrocellulose filters is shown. Species names and developmental times are indicated. Scale bar: 0.5 mm. **(c) **The heat maps represent the patterns of change in standardized mRNA abundance for all the genes in the *D. discoideum *and the *D. purpureum *genomes. Each row represents an average of 85 genes and each column represents a developmental time point (hours). The colors represent relative mRNA abundances (see scale). The genes are ordered according to their regulation pattern in each species. The black lines divide the transcripts, from top to bottom, into: down-regulated, intermediate regulation and up-regulated. The dendrograms represent the differences between the transcriptomes at each time point. **(d) **The maximal similarity between each *D. purpureum *developmental time point (x-axis) to each *D. discoideum *time point (y-axis) across the 7,560 orthologs. The dashed line represents a hypothetical comparison between perfectly synchronous developmental programs.

The dictyostelids are an order of amoebae that prey on bacteria in the soil and propagate by fission as solitary cells. Upon starvation they become social and embark on a developmental program that begins with aggregation of thousands of cells into a mound and ends with a multicellular structure that consists of a ball of spores carried atop a cellular stalk. Despite their vast evolutionary distance, *D. discoideum *and *D. purpureum *exhibit very similar developmental programs and inhabit the same ecological niche [11]. Both organisms begin their multicellular development immediately following starvation, both use chemotaxis towards cAMP as a means of aggregation, and both differentiate into two types of cells during the slug stage - prespore and prestalk cells (Figure 1a). The two cell types eventually develop into a cluster of spores, called the sorus, and a thin rod of vacuolated cells called the stalk. The fruiting bodies of the two species are similar in size and shape [12], although *D. purpureum *commits its cells to the sterile stalk tissue during the multicellular phase by generating a stalk during slug migration, whereas *D. discoideum *does not. There is also a difference in pigmentation of the sori, as illustrated in Figure 1a. Despite the similarities between the species, if cells of *D. discoideum *and *D. purpureum *happen to aggregate together, they soon sort out to form species-specific fruiting bodies [11]. Other prominent differences are a 4-hour delay in aggregation and a 4-hour delay in culmination of *D. purpureum *compared to *D. discoideum*. However, by the end of the 24-hour developmental program, both species have formed fruiting bodies, consisting of spore-filled sori carried atop cellular stalks. We wanted to test whether the developmental transcriptional profiles of the two species mirror the morphological similarities despite the protein sequence divergence.

## Results and discussion

### Conservation of developmental gene expression profiles

We collected RNA samples at 4-hour intervals during the 24-hour developmental programs in two independent replicas for each species and analyzed them by RNA-Seq (Table S1 in the supplementary material [13]). We found that 69% of the *D. discoideum *genome was transcribed, with 12% in unannotated regions. In *D. purpureum*, 74% of the genome was transcribed, with 17% in unannotated regions. The biological replicates were highly similar to each other (mean Pearson's correlation of >0.95 between the biological replicates; Figure S1 in the supplementary material [13]) and the expression of known marker genes was readily validated by quantitative RT-PCR (Figure S2 in the supplementary material [13]). There are 13,970 gene models in *D. discoideum *and 12,410 in *D. purpureum *(R Sucgang *et al*, unpublished work). We found evidence for 8,435 gene transcripts in *D. discoideum *and 9,403 gene transcripts in *D. purpureum *that were expressed at greater than one mRNA molecule per cell (>30 read counts per gene; see Materials and methods) either in growing or in developing cells and had at least 5% mapable sequences. In most cases we found high reproducibility between the transcript levels in the biological replicates (>0.5 Pearson's correlation) but a few groups of genes failed the reproducibility test. One of the interesting groups is a set of heat shock proteins that had coordinate differences in transcript abundance between the biological replicates of *D. discoideum*. We suspect that some of these variable genes represent meaningful responses to subtle differences in the environment, as observed in other systems [14].

Analysis of the biologically reproducible transcripts revealed that the abundance of almost every mRNA changed at least two-fold during development of both species. Figure 1c shows these findings as heat maps with the genes in each species ordered according to their developmental patterns and subdivided into three groups. In *D. discoideum*, 1,779 transcripts are down-regulated, 3,777 are up-regulated, and 2,822 have other patterns of developmental regulation. In *D. purpureum*, 3,168 are down-regulated, 3,472 are up-regulated, and 2,533 have other patterns of regulation. We also compared the similarity between the transcriptomes at each time point using hierarchical clustering and represent the distances between the transcriptomes as dendrograms above the heat maps (Figure 1c). In both species, the largest change in the transcriptome occurs during the transition from unicellularity to multicellularity, between 4 and 8 hours in *D. discoideum *and between 8 and 12 hours in *D. purpureum *(Figure 1c). These results indicate that both developmental programs are accompanied by sweeping changes in the transcriptional regulation of the entire genome and that the major transitions may be conserved.

The genomes of *D. discoideum *and *D. purpureum *contain 7,619 orthologs, more than 50% of the genes in each genome (R Sucgang *et al*, unpublished work). To compare the developmental programs of the two species more closely, we compared the progression of developmental changes in 7,560 orthologs whose transcripts meet our quality criteria. We compared the similarity in the global transcriptional profiles between each *D. purpureum *developmental time point and each *D. discoideum *time point and plotted the maximal correlation (Figure 1d). The results indicate that the general developmental progression is similar between the two species, with two lags in the *D. purpureum *progression relative to *D. discoideum *- one between 4 and 8 hours and another between 16 and 20 hours. The transcriptional delays seen in Figure 1d occur at the same time as the morphological delays seen in Figure 1b, suggesting that the two are causally related.

### Conserved regulation of developmental gene expression

**Figure 2:**
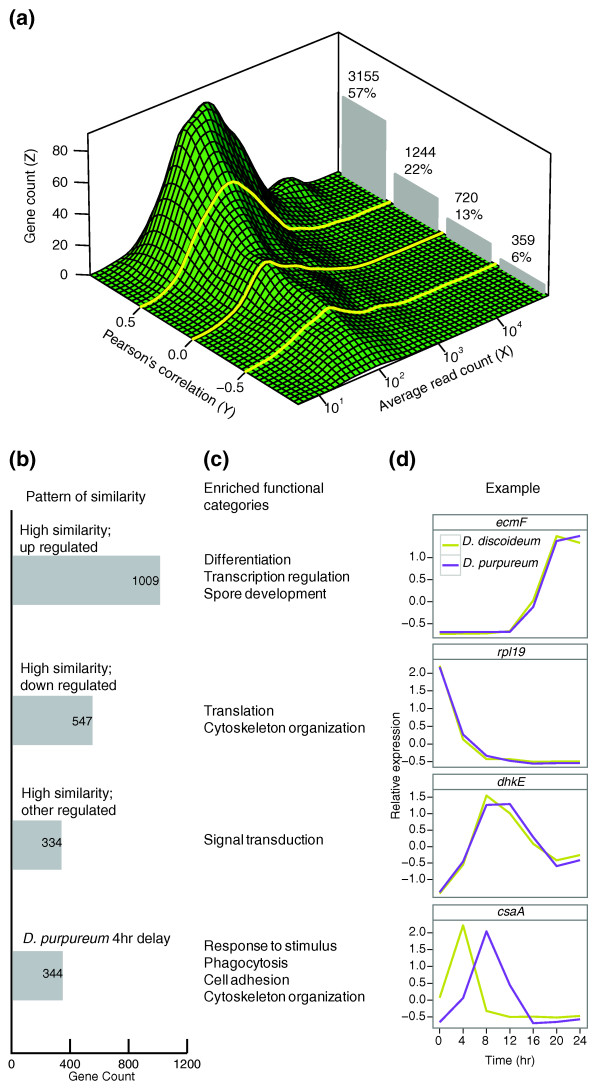
**Conservation of regulation and function between *D. discoideum *and *D. purpureum *transcriptional profiles**. We compared the similarity between the transcriptional profiles of orthologs from the two species. **(a) **The three-dimensional density plot represents the distribution of expression levels (x-axis, average read count) and of the similarities between the transcription profiles of the orthologs (y-axis, Pearson's correlation). The z-axis (gene count) represents the number of genes in each bin (defined by the black gridlines). The histogram behind the density plot summarizes the gene counts in four sections (separated by the yellow lines). The number of genes (top) and their fraction of the total (%) are indicated. **(b) **The bars represent the number of transcripts with various highly conserved expression patterns (gene counts indicated inside bars). **(c) **Prominent Gene Ontology terms enriched within each group. **(d) **Representative expression patterns in *D. discoideum *(yellow) and *D. purpureum *(purple). The time (hours; x-axis), relative mRNA abundance (y-axis), and gene names are indicated.

To quantify the conservation between the developmental transcriptomes of *D. discoideum *and *D. purpureum*, we compared the expression profiles of the orthologs. Figure 2a shows the distribution of expression profile similarities between the two species (Pearson's correlation) and the transcript abundance (average read counts). The three-dimensional density plot indicates that most of the transcripts are similar between the two species, as quantified in the histogram projected on the back panel (Figure 2a). Specifically, the transcriptional profiles of over 57% of the genes are nearly identical (Pearson's correlation >0.5) and another 22% of the genes are similar (Pearson's correlation >0), suggesting that over 75% of the orthologs participate in evolutionarily conserved developmental processes (Figure 2a). Moreover, this transcriptional conservation is not affected by transcript abundance (Pearson's correlation 0.23), as can be seen on the x-axis in Figure 2a. The transcriptional profile of every transcript in *D. discoideum *and *D. purpureum *can be inspected on dictyExpress [15,16].

Coordinate regulation of genes with common functions in specific developmental processes is a good indicator that the functions are being utilized during development [4,17]. We therefore tested which cellular functions are characteristic of the developmentally co-regulated genes. First we determined the maximal similarity between the transcriptional profiles of *D. discoideum *and *D. purpureum *genes with and without temporal transformations. Figure 2 shows four gene groups that exhibit similar patterns of expression between *D. discoideum *and *D. purpureum *(Figure 2b), their enriched biological processes (Figure 2c) and examples of selected gene trajectories (Figure 2d). The enriched annotations among the 1,009 transcriptionally similar (Pearson's correlation >0.75) and up-regulated genes include differentiation, spore development, and regulation of transcription (Figure 2c; Table S2 in the supplementary material [13]). The first two functions suggest that the two species have conserved developmental and differentiation pathways. The latter suggests that regulation of transcription is a central component in developmental regulation, consistent with the finding that most of the genes in the genome are developmentally regulated in both species (Figure 1). The enriched functions among the 547 down-regulated genes include translation (for example, ribosomal proteins), response to bacteria and cytoskeleton organization (Figure 2c; Table S2 in the supplementary material [13]). These functions have central roles in *D. discoideum *growth and our data suggest conservation of these processes in *D. purpureum *[12,18]. We also identified 334 genes with various patterns of developmental regulation, such as transient up or down-regulation, that were enriched in functions related to signal transduction (Figure 2c; Table S2 in the supplementary material [13]), a well-known function in *Dictyostelium *development [12].

Considering the temporal shifts between the developmental programs of *D. discoideum *and *D. purpureum *(Figure 1d), we hypothesized that the expression profiles of orthologous genes required during these stages would be temporally shifted. Therefore, we searched for transcripts that are more similar to each other after applying temporal transformations to the developmental profiles. We found 630 such transcripts, 344 of which exhibit a 4-hour delay in *D. purpureum *compared to *D. discoideum *(Figure 2b). Some of the prominent functions of these transcripts are response to stimulus, phagocytosis, cell adhesion, and cytoskeleton organization (Figure 2c; Table S2 in the supplementary material [13]). Previous studies have shown that these functions are essential during the initiation of development in *D. discoideum *[12,18], so the 4-hour delay in gene expression is consistent with the delayed transition from unicellularity to multicellularity observed in *D. purpureum *(Figure 1b).

We also tested the relationship between the degree of coding sequence conservation and the degree of expression profile conservation, which gave inconsistent results in previous studies [19-21]. Analyzing the orthologous genes between *D. discoideum *and *D. purpureum*, we find no significant correlation between protein sequence conservation and expression profile conservation (Figure S3 in the supplementary material [13]). However, we find that the developmental process is accompanied by a transition from expressing evolutionarily conserved genes to expressing more species-specific genes (Figure S4 in the supplementary material [13]).

### Conserved mRNA abundance

**Figure 3:**
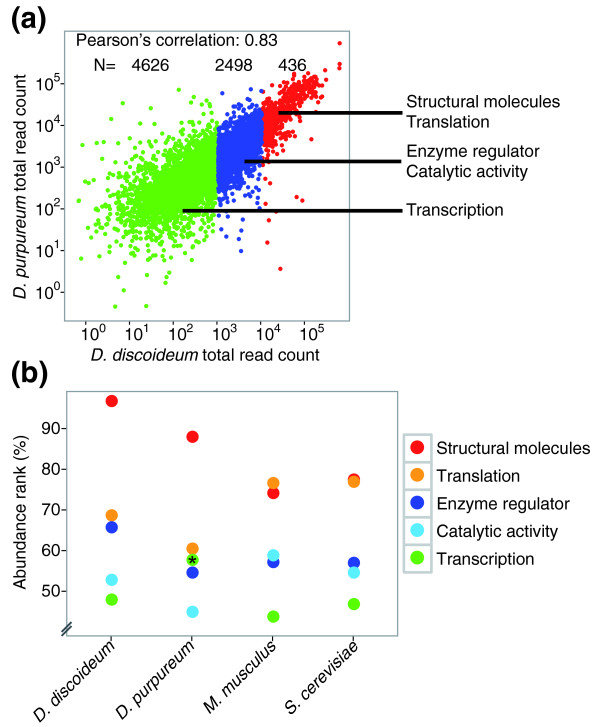
**Conservation of transcript abundance between various species**. **(a) **Scatter plot representing the abundance of the *D. discoideum *transcripts (x-axis, log_10 _scale) compared to their *D. purpureum *orthologs (y-axis, log_10 _scale). Each point represents the sum of read counts over the seven developmental time points. We divided the genes into three groups and indicated enriched Gene Ontology terms. Low abundance, <1,000 reads (green); intermediate abundance, 1,000 to 10,000 reads (blue); and high abundance, >10,000 reads (red). **(b) **We calculated the median gene abundance rank (y-axis, percentile) within five functional categories (indicated by the color code) in amoebae (*D. discoideum *and *D. purpureum*), mice (*M. musculus*), and yeast (*S. cerevisiae*), as indicated (x-axis). The asterisk indicates that only 21 genes represent this category in *D. purpureum *whereas the other species have >100 genes.

Thus far, we have only considered the relative changes in transcript abundance during development in order to focus on gene regulation. RNA-seq data also allow the comparison of transcript abundance between genes within each species and between species. We compared the sums of mRNA abundances from all developmental stages for each of the orthologs and found a surprising similarity between *D. discoideum *and *D. purpureum *(Pearson's correlation = 0.83), suggesting that the absolute mRNA abundances of most genes are conserved between the two species (Figure 3a; Table S3 in the supplementary material [13]). We then divided the transcripts into three groups, based on their abundance, and analyzed the annotations of the genes. We found that mRNAs for structural molecules and for translation (for example, ribosomal proteins) are highly enriched among the 436 most abundant transcripts. The second group (2,498 transcripts) exhibits intermediate transcript levels and is enriched in mRNAs for enzyme regulators and catalytic activity. The least abundant transcripts, which represent over half the orthologs, are enriched in various annotations, including transcription (Table S3 in the supplementary material [13]). These results are consistent with the intuitive notion that transcript abundance correlates with the amount of protein required in the cell. To test the generality of this notion, we compared our data to published RNA-seq data from yeast and mouse [22,23]. We created five broad functional categories using the Gene Ontology (GO) slim terminology [24] and calculated the median gene abundance rank within each category (Figure 3b; Table S4 in the supplementary material [13]). We used ranking rather than actual transcript abundance to allow comparison despite the different normalization methods used in the three studies. In all four species we found that genes involved in translation and in cellular structures had the highest mRNA abundance, transcripts encoding catalytic proteins and enzyme regulators had an intermediate abundance, and mRNAs involved in transcription were among the least abundant ones (Figure 3b). These results highlight the quantitative dimension provided by RNA-seq and show conservation of transcript abundance across large evolutionary distances.

We also analyzed the differences in mRNA abundance between orthologs and non-orthologs in *D. discoideum *and *D. purpureum *and observed that non-orthologous transcripts are less abundant in both species compared to the orthologous transcripts (*t*-test; *D. discoideum P*-value = 3.6e-10; *D. purpureum P*-value = 2.2e-16). This finding is consistent with previous studies showing a positive relationship between sequence conservation and levels of gene expression [25].

### Conservation of cell-type differentiation

Developing *Dictyostelium *cells differentiate into two major cell types - prespore and prestalk. We tested how many genes were cell-type enriched in *D. discoideum *and whether that enrichment was conserved in *D. purpureum*. We separated the prestalk and the prespore cells from the slug stage of *D. discoideum *and *D. purpureum*, and analyzed them by RNA-seq. Previous studies used *in situ *RNA hybridization to identify 132 *D. discoideum *genes that are preferentially expressed in prespore or prestalk cells [26]. We traced the abundance of these transcripts in the *D. discoideum *RNA-seq data and used them as standards to define cell-type enriched transcripts, identifying 850 prespore genes and 915 prestalk genes (Figure S5 and Table S5 in the supplementary material [13]). We then used the *D. purpureum *orthologs of the known *D. discoideum *markers to define cell-type enriched genes in a similar way and identified 1,984 prespore genes and 801 prestalk genes (Figure S5 and Table S6 in the supplementary material [13]). Since we only considered two biological replicas of each species, these data rely on a conservative method for estimating the confidence statistic. A new but less statistically robust method that relies on the sequence coverage of each nucleotide in the transcript yielded quantitatively better results (Figure S5 and Supplementary methods in the supplementary material [13]).

**Figure 4:**
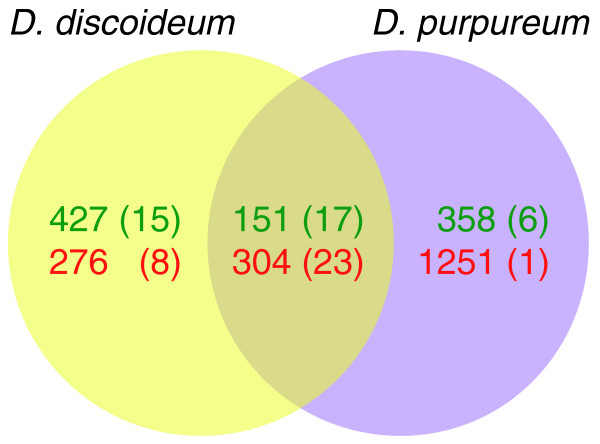
**Conservation of cell-type specificity between *D. discoideum *and *D. purpureum *transcripts**. Similarity between cell-type enriched orthologs. The yellow circle represents *D. discoideum *transcripts, the purple circle represents *D. purpureum*, and the overlap represents the conservation of cell-type-enriched genes. The differentially expressed genes within each set are divided into prespore enriched (green), prestalk enriched (red) and known markers (in parentheses).

We then focused on the 7,560 orthologs and found 1,158 to be cell-type enriched in *D. discoideum *and 2,064 to be cell-type enriched in *D. purpureum*. Of those, 455 transcripts were enriched in the same cell type in both species (Figure 4). This group of conserved cell-type-enriched transcripts was significantly enriched in transcriptionally conserved genes (*n *= 188, hypergeometric *P*-value = 4.5e-7). We hypothesized that the relatively low level of conservation among the cell-type-enriched transcripts was due to the stalk formation during slug migration in *D. purpureum *and not in *D. discoideum*. We therefore traced the expression profiles of the cell-type-enriched transcripts in the developmental transcriptomes to identify prestalk enriched genes that are temporally shifted between the two species, but could not find a significant number within the list of orthologs. The data shown in Figure 4 greatly expand our knowledge of cell-type-enriched transcripts in *Dictyostelium *and indicate that the conservation in the transcriptomes extends to cell type differentiation, albeit to a lesser extent than the developmental conservation.

## Conclusions

The conservation of the developmental transcriptomes of *D. discoideum *and *D. purpureum *is rather surprising, considering the evolutionary distance between the genomes of the two species (R Sucgang *et al*, unpublished work). Previous studies have argued that divergent regulation of gene expression is a major component of morphological divergence during evolution [6,27]. Our analysis shows the other side of that argument, suggesting that conservation of transcriptional regulation may be responsible for anatomical conservation.

Comparison of *D. discoideum *and *D. purpureum *offers a unique insight into the role of transcriptional regulation in developmental programs, because both developmental processes are highly synchronous and the two species have only two major cell types. Furthermore, *Dictyostelium *is particularly amenable to RNA-seq transcriptome analyses since large amounts of homogeneous biological samples can be collected at all stages throughout development and the two major cells types can be separated at the slug stage. Other multicellular organisms may present more complicated patterns of cellular differentiation and it may be difficult to define analogous developmental stages between distant species. Nevertheless, comparative transcriptome analyses by RNA-seq could still be quite informative in such organisms, especially for the analysis of defined tissues and purified cell types.

## Materials and methods

### Growth, development and RNA preparation

For the developmental time courses, we used the *D. discoideum *strain AX4 [28] and the *D. purpureum *strain DpAX1, whose genomes have been sequenced (R Sucgang *et al*, unpublished work) [29]. For cell type enrichment, we used the *D. discoideum *strain NC4 [30] and the *D. purpureum *strain DpAX1. We grew the cells to mid-log phase in association with *Klebsiella aerogenes *bacteria on SM-agar plates [31,32]. To induce development, we collected the cells, washed them as described [31], deposited them on nitrocellulose filters and developed them in the dark at 22°C. At each time point, we collected 1 × 10^8 ^cells directly into 1 ml Trizol reagent (Life Technologies, Carlsbad, CA, USA) and extracted total RNA according to the manufacturer's recommended protocol. We collected cells at the finger stage, prepared prespore and prestalk cells by centrifugation through percoll gradients as described [33], and extracted RNA as above. We repeated each experiment twice, independently. In each case we tested the quality of the RNA by quantitative RT-PCR with oligonucleotides against several known developmental markers (Figure S2 in the supplementary material [13]) and, in the case of cell type enrichment, we tested the RNA by quantitative RT-PCR with oligonucleotides against known cell-type-specific markers from *D. discoideum *[26] and their *D. purpureum *orthologs.

### cDNA preparation

To prepare cDNA, we subjected 20 μg of total RNA to one round of poly-A selection on oligo(dT) beads (Dynal, Carlsbad, CA, USA). We fragmented 125 ng of the resulting RNA to an average size of 200 bases using divalent cations (Fragmentation Buffer, Ambion, Austin, TX, USA) at 70°C for 5 minutes and terminated the reaction with stop buffer (Ambion). We precipitated the fragments by adjusting the reaction to 66 mM NaOAC, pH 5.2, 0.22 mg/ml glycogen and 70% ethanol, washed the precipitate once with 70% ethanol and resuspended it in RNAse free water. We prepared first-strand cDNA with Super Script II reverse transcriptase (Invitrogen, Carlsbad, CA, USA) and 3 μg of random hexamer primers. We then synthesized second strand cDNA with DNA Polymerase I and RNaseH in an Illumina custom buffer (Illumina, San Diego, CA, USA). We purified the products on a QiaQuick PCR column (Qiagen, Valencia, CA, USA) and eluted them in 30 μl EB buffer (Qiagen). We further processed the cDNAs using the Genomic DNA Sequencing Sample Prep Kit (Illumina) according to the manufacturer's recommended protocol. A detailed description of the RNA-seq sample preparation methods is provided in the supplementary material [13].

### Sequencing and data processing

We sequenced the cDNA libraries (read length = 35 bases) on a high-throughput Illumina Genome Analyzer II using the manufacturer's recommended pipeline (versions 1.2 and 1.3). The resulting FASTQ files were mapped in multiple steps using the short-read alignment software novoalign from Novocraft according to the manufacturer's default parameters [34]. First we mapped the reads to the reference genome. Sequenced reads from *D. discoideum *were mapped to the 13 May 2009 genome build of *D. discoideum *from dictyBase [35], while masking the duplicated region of chromosome 2 (nucleotides 3,015,984 to 3,768,555) and a half of the ribosomal DNA palindrome (nucleotides 42,801 to 78,150). Sequenced reads from *D. purpureum *were mapped to the *D. purpureum *genome assembly (R Sucgang *et al*, unpublished work). Sequences that did not match the chromosomal sequences were mapped to a library of all possible splice junctions that we determined using the annotated gene models. The gene models for *D. discoideum *are defined by the 13 May 2009 build from dictyBase [35] and for *D. purpureum *by the published genome annotations (R Sucgang *et al*, unpublished work). Finally, we mapped the remaining RNA-seq reads after trimming two bases from the end of the reads, iteratively, until the reads were shorter than 25 bases. The expanded genome, including the masked chromosomal sequences and all possible splice junctions, and the gene models we used for both species are available in the supplementary material [13]. The nucleotide level coverage can be visualized in the transcriptome browser [36].

### Mapability

We calculated the mapability of every nucleotide by generating all possible 35 bp oligomers from each genome and mapping them back to the respective genome using the default parameters of novoalign [34]. A nucleotide is defined as mapable if the 35 bp sequence starting at that nucleotide can be unambiguously mapped to the genome. We define the effective length of each gene as the count of mapable nucleotides.

### Scaled mRNA abundance levels

In order to compare transcript abundance between different time points and cell types within and between species, we scaled the transcript abundance values to account for mapability and for the total read counts from each sequencing run. Since the coverage across transcripts is variable, we excluded transcripts that are less than 5% mapable. We also excluded transcripts that are not polyadenylated because our library preparation protocol selects for polyadenylated genes. All genes on the mitochondrial or rDNA chromosomes and any tRNA, rRNA or other non-coding RNAs were excluded. We only identified a single ortholog of non-polyadenylated mRNA in the *D. purpureum *genome. We conducted all of the analyses on this filtered list, which consisted of 12,713 *D. discoideum *genes and 12,246 *D. purpureum *genes. We defined the raw abundance level of each transcript (*i*) in a sample (*j*) as the sum of all the unique reads that map to the transcript in the expanded genome. We then scaled this count by the effective gene length and by the total read count from the entire sequencing run as follows:

where *a*_*ij *_is the scaled abundance for all genes *i *from each sample *j*, *r*_*i *_is the sum of reads that mapped to gene *i*, *L *is the median effective gene length of all the genes, *N *is the mean of the total read counts of all the sequencing runs considered in the experiment, *l*_*i *_is the effective length of gene *i *and *n*_*j *_is the total number of uniquely mapped reads from sequencing run *j*, excluding the non-polyadenylated genes. This method accounts for the transcript size, as well as for differences in the total read count between samples, while preserving the dynamic range of the original data. We provide the raw data as well as the scaled data in the supplementary material [13]. We also made the scaled data available for independent exploration through dictyExpress [15,16].

We estimated the number of mRNA molecules per cell as represented by the RNA-seq read count. From each sample of 10^8 ^cells we extracted approximately 500 μg of total RNA. The average transcript length in *D. discoideum *is 1,577 bases and the average molecular weight of a ribonucleotide monophosphate is 339.5 g/mol. Assuming that total RNA contains 4% mRNA [37] (20 μg), we estimated the number of transcripts per cell represented by each RNA-seq read as follows:

Since the initial RNA extraction was from 10^8 ^cells, the number of transcripts per cell is calculated as follows:

Considering an average of 5 × 10^6 ^mRNA reads per RNA-seq lane, we calculated the number of transcripts represented by a sequencing read as:

Each RNA-seq read represents approximately 0.04 transcripts per cell, so 30 reads represent approximately 1 mRNA molecule per cell.

### Statistical analysis

We performed all the statistical analyses in the statistical software package R [38]. The complete analysis presented in the paper can be recreated using the R scripts and the scaled transcript abundance counts provided in the supplementary material [13]. Analyses within each species include all the polyadenylated genes with at least 5% mapable nucleotides, >30 raw read-counts in at least one time point and high reproducibility between biological replicates. For all analyses that require a similarity metric we tested both Pearson's correlation and Spearman correlation. We found little difference between the results and therefore present the results calculated using the Pearson's correlation since it is a more powerful test. We define biologically reproducible genes as those having >0.5 Pearson's correlation between the developmental expression profiles from the two biological replicates. In *D. discoideum*, 795 genes did not have sufficient mapable sequences, whereas in *D. purpureum*, 163 genes failed this criterion. In *D. discoideum*, 715 genes failed the reproducibility criterion and 3,563 were not expressed, whereas in *D. purpureum*, 321 genes failed the reproducibility criterion and 2,522 were not expressed. In *D. discoideum *we also excluded 462 genes that lack a poly-A tail. We identified only one such gene in *D. purpureum*. Comparisons between species only includes the 7,619 identified orthologs between the species (R Sucgang *et al*, unpublished work). All analyses were done on log-transformed scaled read counts.

We defined developmentally up- or down-regulated genes based on the similarity of a gene's trajectory to a hypothetical increasing trajectory using the function y = x, where y is the scaled read count and x is the developmental time point. Genes with >0.5 Pearson's correlation coefficient are defined as up-regulated genes, whereas genes with <-0.5 Pearson's correlation coefficient are down-regulated genes. Invariant genes are defined as having less than a two-fold change in abundance between any two developmental time points.

To identify GO categories enriched within gene lists we used the Cytoscape software version 2.6.3 [39] with the Bingo plugin [40]. Briefly, the tool uses the hypergeometric distribution with a Benjamini and Hochberg false discovery rate correction to identify GO terms found within a gene list more often than expected by chance. The GO annotation files for *Mus musculus *and *Saccharomyces cerevisiae *were obtained from the GO website. The GO files for *D. discoideum *and *D. purpureum *were obtained from dictyBase [35].

### Data visualization

We generated heat maps in Figure 1 with the heatmap.2 function from the gplots package [41]. To allow comparison between gene profiles with different abundances, we normalized the developmental profiles to have a mean of 0 and a standard deviation of 1. The resulting z-scores represent the number of standard deviations a time point is above or below the profile mean and are used to color the heat map. We ordered the genes based on their regulation from down-regulated to up-regulated. To calculate the similarity between time points we performed hierarchical clustering (R function hclust) on the expression vectors from the time points, consisting of all genes, and visualized the results as a dendrogram. We used Pearson's correlation as the distance metric and average linkage as the clustering criterion. In the presentation, objects (individual time points or groups of time points) are joined if they are more similar to each other than to any of the other objects. The vertical distance of the joint from the top is proportional to the dissimilarity between the joined objects.

The three-dimensional visualization in Figure 2 was generated using a two-dimensional kernel density estimation provided in the R package MASS with 50 bins along each dimension [42]. The transcript abundances were calculated as the average of read counts from all developmental stages in both species, and the similarity was calculated using Pearson's correlation between the expression profiles of the orthologs. We divided the distribution into four bins based on the expression profile similarity dimension: >0.5 Pearson's correlation, between 0.5 and 0 Pearson's correlation, between 0 and -0.5 Pearson's correlation, and <-0.5 Pearson's correlation. Genes with <0.75 Pearson's correlation were subjected to various temporal transformations and grouped based on the transformation achieving greater than 0.75 correlation. Using cross-correlation (R function ccf) we determined the temporal shift required for maximal correlation. We grouped genes into four categories: delayed by 4 hours in *D. purpureum*, delayed by >4 hours in *D. purpureum*, delayed by 4 hours in *D. discoideum*, and delayed by >4 hours in *D. discoideum*. The developmental trajectories in Figure 2d were generated by normalization of the expression profiles to have a mean of 0 and standard deviation of 1. The resulting z-scores represent the number of standard deviations a time point is above or below the profile mean.

To measure the similarity of transcript abundance between *D. discoideum *and *D. purpureum*, we created an expression vector consisting of the sum of read counts from all developmental time points for all orthologous genes. We used Pearson's correlation as a measure of similarity between the two expression vectors.

We also compared our data to published mouse and yeast data. We calculated the transcript abundance data for the mouse as the sum of abundances from published data on two replicate samples of brain, liver and muscle transcriptomes [22]. The yeast RNA-seq data are the sum of all the published biological and technical replicates from cells grown in rich media [23]. Since the published data were from different quantification methods, we used transcript abundance ranks rather than straight transcript abundances in comparing the functional categories between the species. We calculated the ranks as follows:

where *P*_*ik *_is the rank (abundance percentile) of category *j *(structural molecule, translation, enzyme regulator, catalytic activity, or transcription) from species *k *(*D. discoideum, D. purpureum, M. musculus, S. cerevisiae*). *g*_*ijk *_is the gene abundance of gene *i *within category *j *within species *k*, and *N*_*k *_is the total number of genes in species *k*. The genes within each category are defined by the GO slim mapping [24].

### Two methods for defining cell-type-specific genes

RNA-seq allows us to define the abundance of each nucleotide and from these values calculate the abundance of genes. There is little technical variability in gene abundance across biological replicates, but at the nucleotide level there is a clear sequence bias that leads to highly variable coverage across a single transcript (and a slight 3' bias; see Figure S6 in the supplementary material [13]). We assessed differential expression of genes using both of these data sets.

### Whole-transcript method

Results derived using the whole-transcript method are shown in Figure S5a,b in the supplementary material [13]. We calculated the differential expression of normalized read counts for each gene using the LIMMA package in R [43]. We fitted a linear model to the log_2_-transformed data with biological replicates and cell types as factors and we used an empirical Bayes method [44] to moderate standard errors. This method does not account for the variability in nucleotide coverage and is limited by the low number of replications. However, we chose to present the results of that method in the figures because it is more commonly used.

### Nucleotide method

We also used the nucleotide coverage in an attempt to account for variability across a transcript and improve the assessment of differential expression. We fitted a linear model using biological replicates and cell types as factors and the log_2_-transformed read counts at each nucleotide across a gene as repeated measurements. This method violates the distributional assumptions of independence, normality and homoscedasticity for linear modeling, but its results are empirically better than the whole-transcript method. Genes with low read counts or bias due to sequence naturally have high variability in the coverage and can only be detected using this type of analysis. The results of using this method and a comparison between the two methods are presented in Figure S5 in the supplementary material [13].

### Defining cell-type enriched transcripts

The cDNA Atlas project defined 132 *D. discoideum *transcripts as cell-type enriched using *in situ *RNA hybridization [26]. We used these data to determine empirical thresholds for defining cell-type enrichment in the RNA-seq data. Since we do not have such data for *D. purpureum*, we used 95 orthologs from the list of 132 *D. discoideum *transcripts to determine the empirical threshold values for *D. purpureum*. We defined differentially expressed genes as those that meet our quality criteria and have at least a two-fold change in abundance between the two cell types and a *P*-value lower than the maximum *P*-value of the known cell-type-specific genes in *D. discoideum*. The list of genes that are differentially expressed using the nucleotide coverage method is a subset of the list of genes found using the gene abundance counts. If we do not impose the minimum read count criteria, many of the genes identified as differentially expressed using the whole-transcript method fall below the 30 read count threshold and therefore had highly variable nucleotide coverage. Using the nucleotide coverage method, this variability is implicitly accounted for within the linear model and low abundance genes are not identified as differentially expressed.

### Data availability

We provide supplement material [13] that includes a downloadable version of all the analyzed data and the R code we used to generate them as well as the supplementary figures and tables referred to in the main text. In addition, we provide a link to a transcriptome browser that allows exploration of all the data through a genome-centric graphical interface as well as detailed data about individual genes and summaries about individual experiments [36], and a link to dictyExpress, allowing exploration and data mining of individual genes and small groups of genes [16]. The raw sequences and mapped data are also deposited in the Gene Expression Omnibus (accession number [GEO:GSE17637]).

## Abbreviations

Bp: base pair; GO: Gene Ontology; RNA-seq: RNA sequencing.

## Authors' contributions

REM, MKK and DF performed the experiments; AP, GR, LZ and TC performed the data analysis; AP, REM and GS wrote the manuscript; all of the authors contributed to the research design, discussed the results and commented on the manuscript.
